# The EZH2–PRC2–H3K27me3 axis governs the endometrial cell cycle and differentiation for blastocyst invasion

**DOI:** 10.1038/s41419-023-05832-x

**Published:** 2023-05-18

**Authors:** Yamato Fukui, Yasushi Hirota, Shizu Aikawa, Akihiko Sakashita, Ryoko Shimizu-Hirota, Norihiko Takeda, Chihiro Ishizawa, Rei Iida, Tetsuaki Kaku, Tomoyuki Hirata, Takehiro Hiraoka, Shun Akaeda, Mitsunori Matsuo, Yutaka Osuga

**Affiliations:** 1 Department of Obstetrics and Gynecology, Graduate School of Medicine, The University of Tokyo, Tokyo, 113-8655 Japan; 2 Department of Molecular Biology, Keio University School of Medicine, Tokyo, 160-0016 Japan; 3 Department of Internal Medicine, Center for Preventive Medicine, Keio University School of Medicine, Tokyo, 160-0016 Japan; 4 Center for Molecular Medicine, Jichi Medical University, Shimotsuke, Tochigi 329-0498 Japan

**Keywords:** Infertility, Disease model, Infertility

## Abstract

Infertility occurs in 15% of couples worldwide. Recurrent implantation failure (RIF) is one of the major problems in in vitro fertilization and embryo transfer (IVF–ET) programs, and how to manage patients with RIF to achieve successful pregnancy outcomes remains unresolved. Here, a uterine polycomb repressive complex 2 (PRC2)-regulated gene network was found to control embryo implantation. Our RNA-seq analyses of the human peri-implantation endometrium obtained from patients with RIF and fertile controls revealed that PRC2 components, including its core enzyme enhancer of zeste homolog 2 (EZH2)-catalyzing H3K27 trimethylation (H3K27me3) and their target genes are dysregulated in the RIF group. Although fertility of uterine epithelium-specific knockout mice of *Ezh2* (eKO mice) was normal, *Ezh2*-deleted mice in the uterine epithelium and stroma (uKO mice) exhibited severe subfertility, suggesting that stromal Ezh2 plays a key role in female fertility. The RNA-seq and ChIP-seq analyses revealed that H3K27me3-related dynamic gene silencing is canceled, and the gene expression of cell-cycle regulators is dysregulated in *Ezh2*-deleted uteri, causing severe epithelial and stromal differentiation defects and failed embryo invasion. Thus, our findings indicate that the EZH2–PRC2–H3K27me3 axis is critical to preparing the endometrium for the blastocyst invasion into the stroma in mice and humans.

## Introduction

Appropriate communication between the fetus and endometrium is critical for healthy pregnancy outcomes [1, 2]. Infertility is an important social concern encountered by approximately 15% of couples during their reproductive age worldwide [3]. In vitro fertilization and embryo transfer (IVF–ET) have been developed in the last 40 years, providing healthy births to infertile couples. However, by improving the techniques to select good-quality sperms, oocytes, and fertilized embryos, only 30% of ETs succeed in having pregnancies [2]. Recurrent implantation failure (RIF) is one of the major issues in IVF–ET programs. Therefore, an inappropriate endometrial condition is believed to be a critical factor to cause RIF [1]; however, how the endometrial dysfunction causes RIF remains unclear.

Embryo implantation is a complex process of communication between blastocysts and endometria, molecularly and physically [1, 4]. Due to similarities in hormonal cycles and the manner of pregnancy maintenance, rodents are often used as a model to analyze embryo implantation processes as an alternative to human studies [1, 2, 4]. After coitus, ovaries provide increasing progesterone (P_4_) levels to prepare the endometria receptive to blastocysts [5]. With the influence of P_4_, the endometrial epithelium stops growing and begins to differentiate [6, 7]. Blastocysts arrive inside the uterine cavity on day 4 of pregnancy (day 1, plug positive day) and attach to the luminal epithelia at midnight on day 4. The epithelial layer surrounding the attached blastocyst creates a crypt deeply carving out the endometria accompanied by epithelial gland extensions [8, 9]. In the same vein, the uterine stromal cells surrounding the blastocysts undergo terminal differentiation and become polyploid cells known as decidual cells, which promotes trophoblast invasion and the following placentation [10]. Any defects in these steps can increase the risk of implantation failures and adverse effects in the following pregnant conditions [1]. Therefore, the endometrial gene networks regulating each event should be clarified during the peri-implantation period to improve pregnancy outcomes.

Gene expressions are generally regulated by transcriptional factors and epigenetic machinery including DNA methylations and histone modifications [11–13]. Methylation(s) on lysine 27 of histone H3 (H3K27me, H3K27me2, and H3K27me3) is a major epigenetic modification to silence gene expressions [13]. Polycomb repressive complex 2 (PRC2) has been known to cause H3K27 methylations that control expressions of critical genes for embryogenesis, organogenesis, and tumorigenesis [14]. It is composed of several molecules, such as Suz12, Eed, Ezh1, and enhancer of zeste homolog 2 (Ezh2). Ezh2 plays a role in the high activity of methyltransferase against H3K27 but requires the presence of Suz12 and Eed for its full activity [15]. In various tissues, single deletion of Ezh2 results in defective cellular functions due to abnormal gene expressions with decreased H3K27me3, indicating that Ezh2 is a core molecule in PRC2 to exert H3K27 methylations [14]. Our recent RNA-seq analysis in the peri-implantation mouse endometria revealed that H3K27me3-targeting genes are highly enriched in differentially expressed genes (DEGs) during implantation transition [16], suggesting the possible roles of PRC2–H3K27me3 in the establishment of early pregnancy.

In this study, we provided evidence for previously unappreciated roles of Ezh2 in the uterus during the peri-implantation period by analyzing human and mouse endometrial tissues. RNA-sequencing of the human peri-implantation endometrium revelaed that EZH2 and PRC2–H3K27me3-targeting genes are more dysregulated in the human endometrium of patients with RIF compared to the fertile controls. To determine the physiological functions of Ezh2 in the endometrium, uterus-specific knockout (KO) mice of *Ezh2* were created. We found that histone modifications by H3K27me3 was impaired by *Ezh2* deletion in the mouse uterus, which increased the gene expression of cell-cycle regulators. Uterine *Ezh2* deficiency also caused the sustained proliferation of the epithelium and reduced terminal differentiation of the stroma, suggesting defective epithelial and stromal differentiation in the *Ezh2*-deficient uterus. Consequently, the abnormal Ezh2–H3K27me3 axis in the endometrium resulted in poor embryo invasion into the stroma, eventually resulting in implantation failure. Our findings indicate an unexplained role of Ezh2 in regulating endometrial differentiation required for blastocyst implantation and the following pregnancy success.

## Results

### EZH2–PRC2–H3K27me3-targeting genes are dysregulated in the human endometrium obtained from women with RIF

**Fig. 1:**
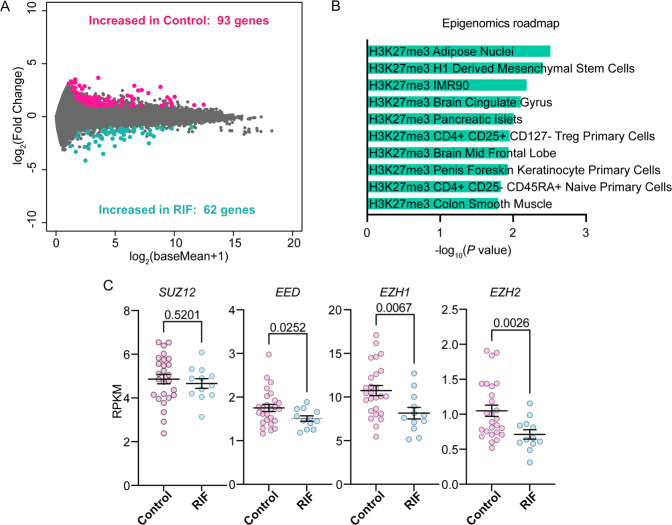
PRC2–H3K27me3-targeting genes are dysregulated in the human endometrium obtained from women with recurrent implantation failure (RIF). **A** Genes showing >twofold difference in expression levels with adjusted *P-*values of <0.5 were evaluated as differentially expressed genes (DEGs). Significantly increased genes (93 genes) in the fertile controls (Control, *n* = 26) are highlighted by magenta, and increased genes (62 genes) in the recurrent implantation failure group (RIF, *n* = 12) are highlighted by turquoise. **B** The upregulated genes in RIF patients were then placed in Enrichr to examine the upstream machinery that regulates gene expressions. H3K27me3-targeting genes are significantly enriched in DEGs. **C** Reads per kilobase of transcript per million mapped read (RPKM) values of major PRC2 components were shown. Among them, *EZH2* was downregulated in the endometrium of the RIF group.

We first investigated the landscape of gene networks that can contribute to a successful implantation outcome in humans. RNA-seq analysis was enrolled in the peri-implantation endometrium obtained from patients with RIF and their fertile controls (Fig. 1A; Table 1). We detected 93 and 62 genes upregulated in the pregnancy and RIF group, respectively (Fig. 1A) (Data S1). These DEGs were then placed in Enrichr to examine upstream machinery that regulates gene expressions. H3K27me3-targeting genes were found to be significantly enriched in the upregulated genes in the RIF group, suggesting H3K27me3-induced gene suppression is critical for human embryo implantation (Fig. 1B). Further analysis showed read per kilo million (RPKM) values in major PRC2 components (Fig. 1C). *EZH2*, as well as *EED* and *EZH1*, were found to be downregulated in the endometrium of the RIF group (Fig. 1C). These data indicate that PRC2 is involved in making the endometrium receptive through histone modifications by H3K27me3. Among the PRC2 components, we focused on EZH2, a crucial enzyme to catalyze H3K27 trimethylations [14, 17]. EZH2 immunostaining by the Human Protein Atlas provides higher expressions in the endometrium of women of reproductive ages than those of advanced ages (Fig. S1), also indicating possible roles of EZH2 in female reproduction.

**Table 1:** Clinical information of patients tested in Fig. 1.

	Control	RIF	*P*-value
Number of patients	26	12	n/a
Age (years, mean ± SD)	35.0 ± 3.2	36.2 ± 2.0	0.19^a^
Patients experienced previous pregnancy: *N* (%)	10 (38.5)	6 (50)	0.72^b^
Patients experienced previous delivery: *N* (%)	4 (15)	4 (33)	0.2^b^
Number of previous embryo transfers per patient: median (IQR)	3 (2-4)	2 (2-3)	0.055^c^

For each parameter, statistical analyses were employed between the RIF and fertile control groups. n/a not applicable.

^a^Student’s *t* test.

^b^Fisher’s exact test.

^c^Mann–Whitney *U* test.

### Deficiency of uterine Ezh2 compromises early pregnancy

**Fig. 2:**
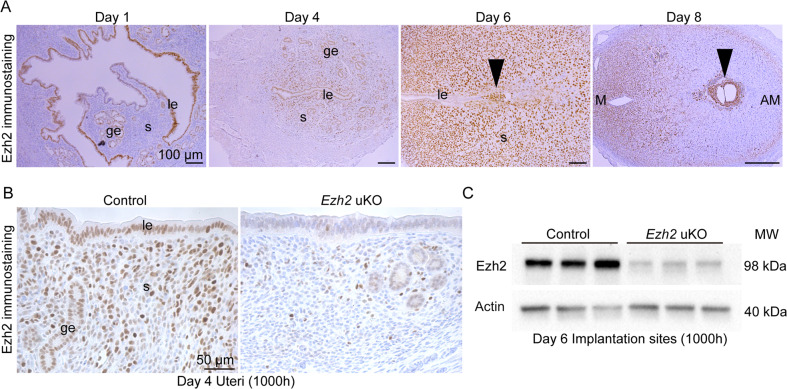
*Ezh2* is highly expressed in the mouse endometrium during early pregnancy. **A** Spatiotemporal Ezh2 expressions in mouse pregnant uteri. Each image is representative of at least three samples. Scale bar = 100 μm; arrowhead, embryo; le, luminal epithelium; ge, glandular epithelium; s, stroma; M, mesometrial pole; AM, anti-mesometrial pole. **B**, **C** The efficient deletion of Ezh2 was confirmed by immunostaining on day 4 (**B**) and Western blotting on day 6 (**C**). **B** Scale bar = 50 μm; le luminal epithelium, ge glandular epithelium, s stroma. At least three mice were tested for each group.

Results of human endometria encouraged us to pursue EZH2 functions during pregnancy. Ezh2 immunolocalization in the mouse uteri was examined during early pregnancy (Fig. 2A). Ezh2 was highly expressed in the luminal epithelium on day 1 of pregnancy when the vaginal plug was visible. Stromal expression of Ezh2 was increased on day 4 and becomes more evident on day 6 when its epithelial expression was decreased. On day 8, stromal Ezh2 was intense on the mesometrial pole (M pole) but was poor around the embryos. Thus, Ezh2 was found to be highly expressed in the mouse endometrium during early pregnancy.

**Fig. 3:**
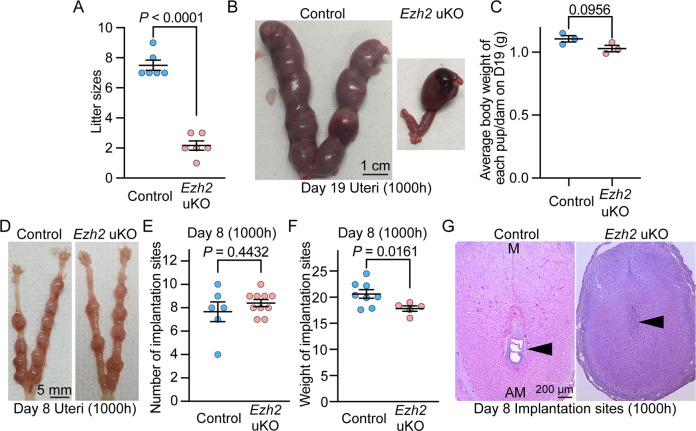
Mice missing uterine *Ezh2* show subfertility due to implantation failures. **A** The litter size was decreased in *Ezh2* uKO (*n* = 6 independent dams) compared with their littermate controls (*n* = 6). Data are presented as mean ± SEM, and *P*-value was provided using Student’s *t*-test. **B** Representative images of pregnant uteri from *Ezh2* control and uKO on day 19 of pregnancy. At least four mice were assessed for each group. **C** The average body weight of each pup per dam from *Ezh2* control and uKO on day 19 of pregnancy. Three dams were assessed for each group. **D** Representative images of pregnant uteri from *Ezh2* control and uKO on day 8 of pregnancy. At least four mice were assessed for each group. **E** The average number of embryo implantation sites per uterus on day 8 of pregnancy: *n* = 6 for the control and *n* = 10 for *Ezh2* uKO. Data are presented as mean ± SEM, and *P*-value was provided using Student’s *t*-test. **F** The average weight of each implantation site per uterus on day 8 of pregnancy: *n* = 8 for control and *n* = 5 for *Ezh2* uKO. Data are presented as mean ± SEM, and *P*-value was provided using Student’s *t*-test. **G** H&E staining showed embryo resorption in *Ezh2* uKO. Scale bar = 200 μm; arrowhead, embryo; M mesometrial pole, AM anti-mesometrial pole. At least *n* = 3 individual tissues were tested for each genotype.

To investigate the roles of uterine Ezh2, uterine deletion of *Ezh2* (*Ezh2* uKO) was initiated in mice by crossing *Ezh2*-floxed mice with those carrying a *Pgr*-Cre driver. Efficient deletion of Ezh2 protein in *Ezh2* uKO was confirmed by immunostaining (Fig. 2B) and Western blotting (Fig. 2C). To examine reproductive phenotypes of *Ezh2* uKO and *Ezh2*-floxed females (*Ezh2* control), we mated them with fertile wild-type (WT) male mice. The litter size was significantly lower in *Ezh2* uKO than in the controls (Fig. 3A). Moreover, *Ezh2* uKO demonstrated reduced numbers of implantation sites on day 19 of pregnancy (Fig. 3B), but without significant differences in body weight (Fig. 3C). On day 8, the numbers of implantation sites (ISs) were comparable between the two groups (Fig. 3D, E). However, the weight of each IS and histological analyses showed that embryonic development is markedly impaired in the implantation sites of *Ezh2* uKO on day 8 (Fig. 3F, G), suggesting that the embryo implantation process earlier than day 8 is abnormal in *Ezh2* uKO.

### Deficiency of uterine Ezh2 impairs embryo implantation

**Fig. 4:**
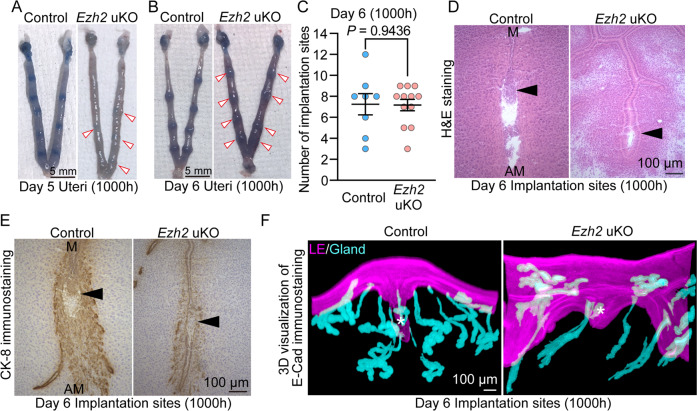
Abnormal implantation chambers in *Ezh2*-deleted uteri. **A** Representative images of *Ezh2* uKO on day 5 of pregnancy. Arrowheads indicate faint blue bands: *n* = 14 for control and *n* = 16 for *Ezh2* uKO. **B** Representative images of *Ezh2* uKO on day 6 of pregnancy. Arrowheads indicate blue bands. **C** The average number of implantation sites in (**B**): *n* = 8 for control and *n* = 12 for *Ezh2* uKO. Data are presented as mean ± SEM, and *P*-value was provided using Student’s *t*-test. **D** H&E staining showed abnormal embryo implantation sites in *Ezh2* uKO on day 6. Scale bar = 100 μm; arrowhead, embryo; M mesometrial pole, AM anti-mesometrial pole. **E** Representative images of cytokeratin 8 (CK-8, a marker for epithelial and trophoblastic cells) showed poor invasions of trophoblast cells into *Ezh2*-deleted stroma on day 6. Scale bar = 100 μm; arrowhead, embryo; M mesometrial pole, AM anti-mesometrial pole. **F** Three-dimensional (3D) landscapes of the uterine epithelia in embryo implantation sites from the control and *Ezh2* uKO uteri on day 6: *n* = 3 for each genotype. Scale bar = 100 μm; *, embryo. For sectional images, at least three individual tissues were tested.

We then traced back the earlier pregnancy events. In normal uteri, embryo attachment increases vascular permeabilities in the stroma in the vicinity of attached embryos, which can be visualized by intravenously injecting blue dye on the morning of day 5 onward [18]. As control uteri had clear blue bands on the morning of day 5, *Ezh2* uKO showed very faint blue reactions with reduced number of ISs at this stage (Fig. 4A). Blue reactions became comparably evident in both genotypes on the morning of day 6 (Fig. 4B, C), whereas abnormal ISs were observed in *Ezh2* uKO by histological analyses (Fig. 4D–F). The numbers of ISs demarcated by blue reactions were decreased in *Ezh2* uKO on day 5 but not on day 6, suggesting a delayed timing of embryo attachment in *Ezh2* uKO (Fig. 4A, B). In hematoxylin and eosin (H&E) staining, *Ezh2* controls showed elimination of epithelial alignment surrounding well-enlarged embryos, a typical morphology of ISs on day 6 (Fig. 4D) [19–21]. Conversely, small blastocysts existed within the intact luminal epithelial layer in *Ezh2* uKO (Fig. 4D).

We previously showed that failed epithelial removal prevents embryo invasion [19, 20]. Similar to those reports, trophoblast invasion was aborted in *Ezh2* uKO as shown by CK-8 immunostaining (Fig. 4E). Three-dimensional landscapes of uterine epithelia were also examined in embryo ISs. Recent studies revealed that the appropriate formation of the epithelial chamber surrounding the embryos is a key step to ensure healthy pregnancy outcomes [8, 9, 22]. In contrast to the deep epithelial crypts and well-extended glands in the *Ezh2* control, shallow crypts and underdeveloped glands were observed in *Ezh2* uKO (Fig. 4F). Collectively, these results demonstrated the roles of *Ezh2* in the appropriate embryo implantation.

### Endometrial functions are appropriately regulated by uterine Ezh2, but independent of uterine epithelial Ezh2

**Fig. 5:**
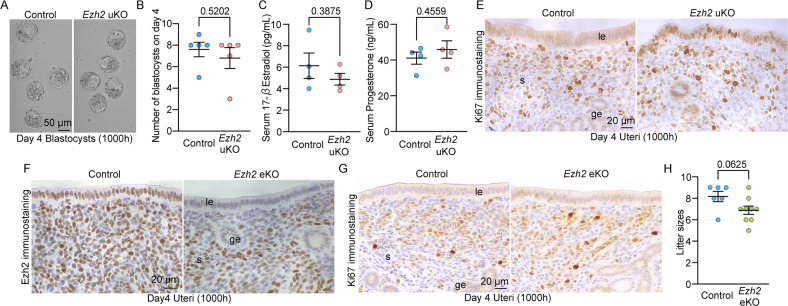
Proliferation-differentiation switching is flawed in *Ezh2* uKO, but not in *Ezh2* eKO. **A**, **B** Both the morphology and number of blastocysts were normal in *Ezh2* uKO on day 4. Scale bar in **A**: 50 µm. In **B**, data are shown as mean ± SEM, and *P*-value was provided using Student’s *t*-test: *n* = 5 mice were tested for each group. **C**, **D** Serum E_2_ (**C**) and P_4_ (**D**) on day 4 of pregnancy were comparable between *Ezh2* uKO and their littermate controls. Data are presented as mean ± SEM, and *P*-values were provided using Student’s *t*-test: *n* = 4 mice for each group. **E** Representative images of Ki67 immunostaining in the uteri on day 4. **F** Representative images of Ezh2 immunostaining in control and *Ezh2* eKO uteri on day 4 of pregnancy. Scale bar = 50 μm; le luminal epithelium, ge glandular epithelium, s stroma. **G** Representative images of Ki67 immunostaining in the uteri on day 4. At least three mice were tested for each group. **H** Scattered plots of average litter sizes from the control (*n* = 6) and *Ezh2* eKO (*n* = 9) dams. Data are presented as mean ± SEM, and *P*-value was provided using Student’s *t*-test. For each immunostaining data, at least three individual tissues were tested.

Defective implantation in *Ezh2* uKO made us suspect the effects on uterine receptivity. In mice, blastocysts enter the uterine cavity on the morning of day 4 and completely attach to the luminal epithelia on the midnight of day 4. We detected corresponding numbers of hatched blastocysts in each genotype (Fig. 5A, B), indicating that uterine Ezh2 does not influence embryo development before attachment. We also tested serum levels of female hormones P_4_ and E_2_ which are critical for the establishment of pregnancy [4, 23]. However, no evident difference was observed in these female sex hormones, showing a comparable ovarian function between each genotype (Fig. 5C, D).

Previously, our and other studies reported that proliferation-differentiation switching (PDS) is a critical determinant for implantation success [4, 6, 20]. At post-ovulation, the epithelial cells develop under the influence of E_2_, and thus, elevated levels of circulating P_4_ stop the epithelial growth and promote stromal proliferation [4, 23]. Defective endometrial functions such as reduced responses against female sex hormones [6, 24, 25] and cell-cycle regulations [20, 26] cause failed embryo implantation and pregnancy termination in later stages. Ki67 immunostaining revealed sustained epithelial proliferation in *Ezh2* uKO, indicating that the embryo receptivity is compromised by *Ezh2* deletion (Fig. 5E). Previous studies have identified possible roles of uterine epithelial Ezh2 on proliferation as abnormal epithelial expansions occur by uterine *Ezh2* deletion [27, 28]. However, normal PDS was observed in mice with uterine epithelium-specific *Ezh2* deletion (*Ezh2* eKO), although efficient *Ezh2* deletion was confirmed in the endometrial epithelium (Fig. 5F, G). Furthermore, our study demonstrated that *Ezh2* eKO females were fertile with normal litter size, suggesting the increased impact of stromal Ezh2 on endometrial functions during pregnancy. Our notion of Ezh2-regulated PDS is also supported by RNA-seq analysis of the mouse uteri on day 4. A total of 156 DEGs were detected in *Ezh2*-missing uteri (Fig. S2A; Data S2). Considering that Ezh2-catalyzing H3K27 methylation silencing gene expressions [11, 17], we especially focused on upregulated genes in *Ezh2* uKO. Interestingly, these increased genes had high enrichment on cell proliferation (e.g., PI3K-Akt, Hippo, and p53 signaling) and estrogen signaling pathways (Fig. S2B). Consistent with this result, levels of E_2_-responsive genes were found to be significantly increased despite normal levels of P_4_-regulated genes in *Ezh2* uKO (Figure S2C). Collectively, these results showed that stromal Ezh2, rather than epithelial one, regulates gene expressions important for the endometrial PDS in the early pregnancy.

### Endometrial Ezh2 suppresses cell-cycle-related genes by H3K27 trimethylation during decidualization processes

**Fig. 6:**
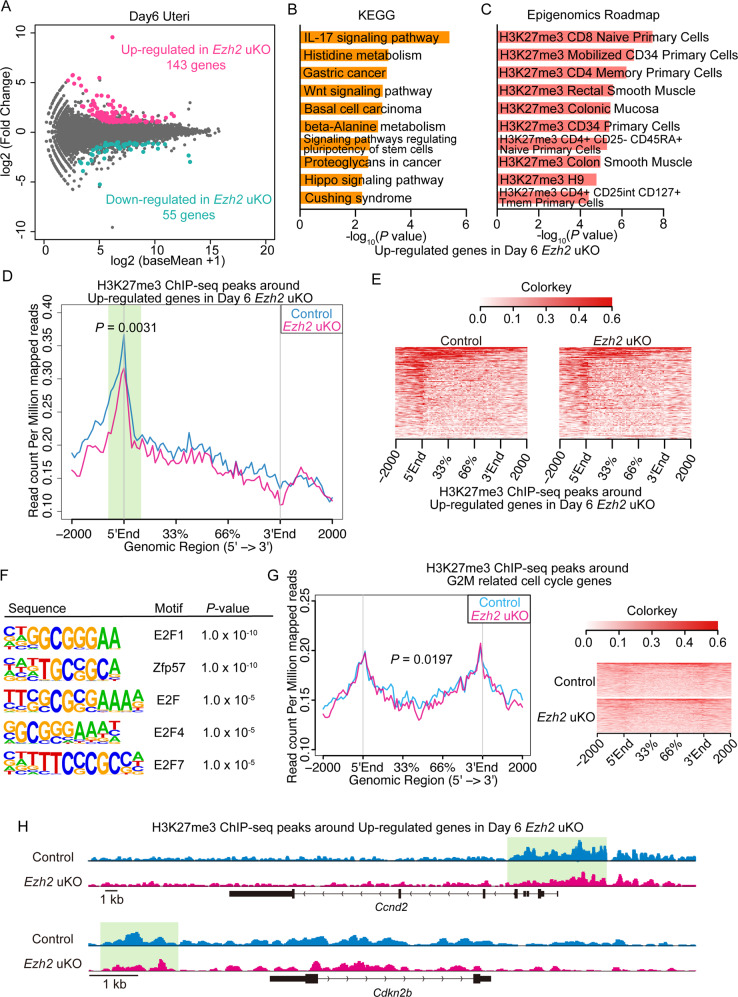
Endometrial Ezh2 suppresses cell-cycle-related genes by H3K27 trimethylation during decidualization processes. **A** MA-plot of RNA-seq results comparing implantation sites on day 6 from the control or *Ezh2* uKO females. Genes with log2-fold change (FC) > |1| and adjusted *P*-value of <0.05 are marked in red (upregulated genes) or blue (downregulated genes): *n* = 3 for each genotype. **B**, **C** Bar graphs depicting enriched KEGG pathways (**B**) and epigenomics roadmap (**C**) in upregulated genes in *Ezh2* uKO on day 6. **D** Average profiles of H3K27me3 on genomic regions of upregulated genes (Fig. 6A) in *Ezh2*-deleted uteri on day 6 (*P* = 0.0031 by Mann–Whitney *U* test): *n* = 2 for each genotype. **E** Heatmaps depicting reduced H3K27me3 on genomic regions of upregulated genes in *Ezh2*-deleted uteri on day 6. **F** HOMER motif analyses of H3K27me3 peaks in day 6 endometria for putative TF binding sites. **G** Average profiles (left) and heatmaps (right) depicting reduced H3K27me3 on genomic regions of G2/M related genes in *Ezh2*-deleted uteri on day 6 (*P* = 0.0197 by Mann–Whitney *U* test): *n* = 2 for each genotype. **H** Representative track views of H3K27me3 around the upregulated genes (*Ccnd2* and *Cdkn2b*) in *Ezh2*-deleted uteri on day 6. Green-highlighted regions demonstrate that uterine deletion of Ezh2 compromises H3K27me3 modification around TSSs and 5′ upstream of each gene.

Recent studies implicated that defective stromal functions increase the risk of failures in receptivity, decidualization, embryo invasions, and following pregnancy maintenance [6, 20, 29]. Considering that decidual reactions were compromised in *Ezh2* uKO (Fig. 4) and PDS was also impaired in this milieu independently on epithelial Ezh2 (Fig. 5), stromal Ezh2 seems more critical to secure pregnancy outcomes. This notion prompted us to resolve how Ezh2, a histone methyltransferase, regulates endometrial functions in the stroma. To investigate gene expression landscapes governed by Ezh2, endometrial tissues were collected on day 6 when decidual reactions become evident in the stroma [29]. RNA-seq analysis revealed a total of 198 DEGs in *Ezh2*-deleted endometria (Fig. 6A; Data S3). As with the upregulated genes on day 4 (Figure S2B), cell proliferation-related KEGGs were enriched on day 6-expressed genes in *Ezh2* uKO (Fig. 6B). Notably, upregulated genes have manifested enrichments in H3K27me3-targeting genes (Fig. 6C), indicating that Ezh2 directly silences these genes through histone methylations during the decidualized stage. We also noted that long non-coding RNAs (lncRNAs) were included in the up-regulated genes in *Ezh2* uKO on day 6 (Fig. S3, Data S3). Among the 5 upregulated lncRNAs, *H19* and *Hotair* have been reported as upregulation as well as scaffolds for Ezh2 in cancer cells [30–32]. There would be functional compensation against missing Ezh2 expression in the uterus. Chromatin immunoprecipitation (ChIP)-seq analysis targeting the H3K27me3 showed the significantly decreased signals around the upregulated genes, especially on their transcription start sites (TSSs) (Fig. 6D, E). Motif analysis also revealed H3K27me3 peaks were significantly enriched on the binding motifs for cell proliferation related transcriptional factors (TFs) such as E2F1 and E2F4 (Fig. 6F). Furthermore, Ezh2-deleted endometrial tissues exhibited reduced levels of H3K27me3 peaks around G2M-related genes (Fig. 6G). Cell-cycle-related genes, *Ccnd2* and *Cdkn2b*, both upregulated in *Ezh2*-missing endometria, were especially examined on day 6 (Data S3). Peak visualizations around these two genes revealed that H3K27me3 modifications on 5’-genomic regions decreased by *Ezh2* deletion (Fig. 6H). In summary, our analyses demonstrate that endometrial Ezh2 suppresses cell-cycle-related genes by H3K27 trimethylations in the decidualized endometria.

### Ezh2-deleted stroma fails to undergo terminal differentiation into the decidual cells

Cell cycles are strictly controlled by various cyclins and their related molecules [33, 34]. Cell-cycle phases are categorized into G1, S, G2/M, and G0 based on the status of DNA synthesis and mitosis in cells. Cyclins are phosphorylated by their upstream kinases, undergoing activation to specific cell-cycle stages. Dysregulation of these molecules results in increased cell proliferation and failed cell mitosis as commonly observed in tumorigenic situations [34]. Deciduae are formed by differentiating stromal cells around the embryos, exhibiting a unique character with polyploidy although decidualization is not a pathological condition [10, 33]. CDK4/6 in the uterine stroma has been found important for decidual reactions by activating cyclin D (Ccnd) molecules to run G1 to S phases [33]. CDK6 also promotes repeating cycles between S and G2 phases, making stromal cells polyploid. Cyclin-D kinase inhibitor (CDKN) molecules can help cells move to the M phase for cell divisions but are suppressed in decidual cells [33].

**Fig. 7:**
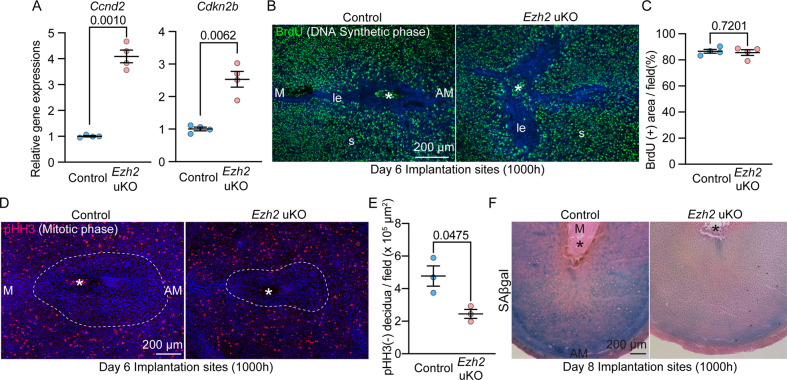
Ezh2-deleted stroma fails to undergo terminal differentiation into matured decidual cells. **A** RT-qPCR validations of *Ccnd2* and *Cdkn2b*, which were upregulated in *Ezh2* uKO due to reduced H3K27me3: *n* = 4 for each genotype. **B**, **C** Representative images of immunofluorescence (IF) of BrdU (an S-phase marker) and BrdU (+) area per field quantified by Image J (**C**) in the uteri on day 6. Four individual implantation sites were tested for each genotype. Scale bar = 200 μm (**B**); *, embryo; M mesometrial side, AM anti-mesometrial side. **D**, **E** Representative images of phospho-histone H3 (pHH3) (a mitotic cell marker) (**D**) and pHH3 (−) decidual area in the uteri on day 6. Three individual implantation sites were tested for each genotype. Scale bar = 200 µm (**D**); *, embryo; M mesometrial side, AM anti-mesometrial side. **F** Flawed terminal differentiation of the uterine stroma in *Ezh2* uKO as depicted by reduced senescence-associated β-galactosidase (SAβgal) activity in the decidua on day 8 of pregnancy: *n* = 5 individual implantation sites were tested for each group. Scale bar = 200 μm; *, embryo; M mesometrial pole, AM anti-mesometrial pole.

**Fig. 8:**
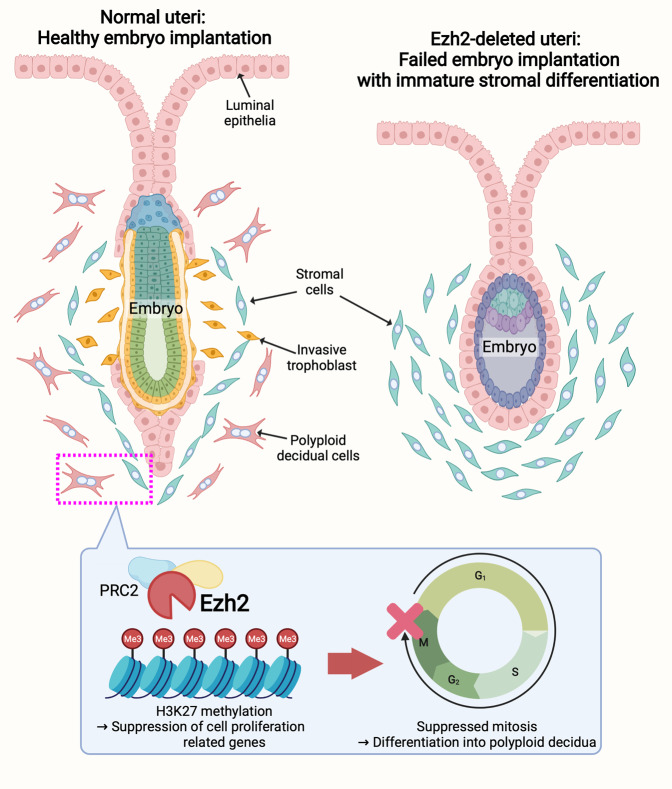
Graphical summary of Ezh2 role in the endometrium during embryo implantation. In the normal uterus, Ezh2 is highly induced in the stroma during embryo attachment. Ezh2 catalyzes H3K27 methylations targeting cell proliferation-related genes, inhibiting mitosis, and facilitating stromal differentiation into the polyploid decidua. In *Ezh2*-deleted uteri, stromal cells with high proliferation activity fail to differentiate into matured decidual cells, eventually compromising the embryo implantation and following pregnancy maintenance. The image was created at BioRender.com.

In agreement with RNA-seq and ChIP-seq results, qPCR also showed significant *Ccnd2* and *Cdkn2b* increases in *Ezh2*-deleted uteri (Fig. 7A). These data allowed the identification of the effects of upregulated cell-cycle genes on decidual reactions. To investigate cell-cycle conditions in the endometrium of *Ezh2* uKO, cells with BrdU that mark S-phase cells were traced (Fig. 7B, C). In the same vein, phospho-histone H3 (pHH3), a mitotic cell marker, was also stained (Fig. 7D, E). As both the control and *Ezh2* uKO had a high content of BrdU-positive cells in the stroma (Fig. 7B, C), pHH3-positive cells were more enriched in the *Ezh2*-deleted stroma surrounding the embryo (Fig. 7D, E). We previously reported that fully differentiated decidua exhibit senescence-related phenotypes; hence, they are depicted by senescence-associated (SA) β-galactosidase (SAβgal) staining [35, 36]. An obvious reduction was detected in SAβgal staining of the *Ezh2*-deleted stroma (Fig. 7F), providing evidence that Ezh2 is critical for terminal differentiation of uterine stroma. Therefore, our data demonstrate that Ezh2 regulates stromal differentiation into functional decidua by silencing cell-cycle-related genes (Fig. 8).

## Discussion

Maternal nidation environments are key determinants for developing embryos to ensure their healthy growth [1]. Embryo implantation anomalies have been reported to severely affect pregnancy maintenance spanning species including humans [2]. Most of the studies using KO mice have shown that several single molecules influence the implantation and following pregnancy conditions, whereas their impacts in humans remain largely uncertain [1, 4]. The recent development of next-generation sequencing found that gene expressions are dynamically altered during menstrual cycles, possibly contributing to endometrial regenerations and implantation window opening [37, 38]. Although this beneficial information regarding menstrual tissues has already been reported, it remains unclear how gene expression signatures differ between endometria with successful and failed implantation. In our study, we dissected DEGs in human peri-implantation endometrium obtained from RIF and fertile patients. Enrichments of PRC2–H3K27me3-targeting genes were evident in DEGs, implying the possible roles of this axis to regulate genome-wide expressions during embryo implantation. Furthermore, the essential roles of Ezh2 in early pregnancy were identified by applying next-generation sequencing in *Ezh2* uKO endometria. Previously, Nanjappa et al. and Fang et al. reported that *Ezh2* uKO uteri exhibit abnormal epithelial integrities in their old ages; however, detailed mechanisms were not shown [27, 28]. They also compared gene expressions between the control and *Ezh2* uKO under the treatment of exogenous P_4_ and E_2_, which might explain increased epithelial cell expansions in this milieu [39]. Recently, Osokine et al. investigated how Ezh2 contributes to decidual functions upon wound healing [40], focusing on post-implantation events after day 8 of implantation when deciduae were already terminally differentiated [36]. However, it remained obscure the impact of uterine Ezh2 against embryo implantation which is the initial stage of reciprocal communications between the embryo and the endometrium. In contrast to these previous studies, we investigated uteri in *Ezh2* uKO during the peri-implantation period, and newly found that uterine Ezh2 controls epithelial and stromal differentiation and blastocyst invasion into the stroma. Combined with analysis of human endometrium obtained from RIF patients, our study uncovered a novel role for uterine Ezh2 in embryo implantation.

In contrast to the previously highlighted epithelial roles of Ezh2 [27, 28], epithelial-specific deletion of Ezh2 was found to not affect female fertility, suggesting that stromal Ezh2 indirectly regulates epithelial functions. Similar epithelial–mesenchymal interactions have been reported by this and other studies: Pgr, Hmgb1, Hif2a, and Rb1 [6, 19, 20, 41]. The importance of stromal PRC2 was noted in human pathology [14, 42, 43]. Genetic mutations of SUZ12, a PRC2 component supporting EZH2 activation, are highly detected in the endometrial stromal sarcoma with increased stromal proliferation [42, 43]. As mutations in PRC2 components are often observed in tumorigenic conditions with high cell proliferation potencies [14], our observations in this study can be applied to other tissues than the pregnant uteri.

To the best of our knowledge, this is the first study that evaluated the EZH2–PRC2–H3K27me3 axis in patients with RIF. We revealed that molecules associated with PRC2 and H3K27me3 functions are dysregulated in the human peri-implantation endometrium of patients with RIF than fertile controls. Notably, EZH2 was downregulated in a more diverse human population compared to a well-controlled mouse model. Our previous studies have demonstrated that P_4_ signaling is critical for endometrial receptivity by regulating PDS in endometrial cells [20, 23], which was also observed in our *Ezh2* uKO model. Interestingly, we previously observed the impairment of PDS in human RIF patients [20], indicating that disturbed P_4_-PDS signaling in the milieu. In addition, there are reports that P_4_ upregulates the EZH2 expression in various human tissues [44, 45]. In this study, all human specimens were obtained in the peri-implantation period on day 7 of P_4_ administration from women under the same protocol of hormonal replacement treatment used for the frozen embryo transfer. Taken all together, it is possible that EZH2 expressions in human RIF patients was reduced due to poor P_4_ responsiveness. Our findings indicate that dysregulated genes associated with PRC2 and H3K27me3 functions are endometrial RIF biomarkers, although further research is required to assess whether these markers are clinically useful to diagnose RIF.

While we demonstrate the importance of Ezh2 in early pregnant uteri, it remains unclear how PRC2 can target specific gene regions crucial for endometrial functions. A previous study reported that hypomethylations on specific loci can weaken PRC2 binding to its target promoters [46]. Another possible mechanism is governed by PRC1, which is composed of multiple molecules to ubiquitinate lysine 119 on histone H2A (H2AK119u1) [47, 48]. PRC1-induced H2AK119u1 can recruit PRC2 to promote H3K27 methylations [47]. As pharmaceutical inhibitions of either DNA methylations or PRC1 resulted in terminations of pregnancy in mice [49, 50], these epigenetic regulations may prepare uterine genomes for the following PRC2 binding. We also have not yet assessed how PRC2-induced H3K27me3 is canceled during the pregnancy progression. A report demonstrated that uterine tissues exhibit acetylation on lysines 4 and 27 of histone H3, the two major active histone markers, upon parturition [51]. This implies that some system works to determine pregnancy stages, switching epigenetic modifications. Future endeavors should examine how uterine cells respond to pregnancy conditions with appropriate gene expressions at each pregnancy condition.

## Methods

### Collection of human endometrial tissues in the peri-implantation period

Human endometrial tissues in the peri-implantation period were obtained from patients undergoing IVF–ET treatment aged under 40 years. Endometrial biopsy was performed as previously described [20]. The specimens obtained from those with uterine fibroids, adenomyosis, endometrial polyps, endometrial hyperplasia, and endometriosis were excluded from the study because these diseases have the possibility to affect endometrial receptivity. To minimize individual differences in hormonal status, the same protocol of hormonal replacement cycle for frozen ET was used for all patients in the cycle of endometrial biopsy. Endometrial biopsies were performed on day 7 of P_4_ administration during a hormonal replacement cycle, which is considered the peri-implantation period in humans. Patients underwent ET in subsequent cycles after the endometrial biopsy, and the outcome of clinical pregnancy was monitored. RIF patients were defined as those who had more than two failed embryo transfer cycles using good-quality embryos. The fertile controls were defined as patients who had clinical pregnancy in the subsequent cycle after the endometrial biopsy. Twelve and 26 independent endometrial samples were collected from patients with RIF and fertile controls, respectively. Table 1 demonstrated the information of the patients with RIF and the fertile controls. The study protocols using human specimens were approved by the institutional review board of the University of Tokyo (IRB numbers: 10991 and 2019241 G), and each woman signed informed consent for the use of tissues.

### RNA-seq of the human endometrium

RNA extraction from human endometrial tissues was performed using the RNeasy Plus Mini Kit (Qiagen, Hilden, Germany). RNA-seq was performed at Macrogen Japan (Tokyo, Japan) on 38 specimens: 26 patients clinically pregnant as a result of embryo transfers after endometrial biopsy (fertile controls; successful implantation group) and 12 who did not become clinically pregnant (the RIF group; failed implantation group). The resulting raw read files were aligned on the human genome sequence (GRCh38/hg38) using the Hisat2 version 2.1.0 [52], and the number of reads at each locus was counted using the featureCount function [53] in the Subread tool. To compare expression levels between successful and failed implantation samples, read count files were submitted to DESeq2 version 1.16.1 [54]. DEGs were defined as genes showing >twofold difference in expression levels with a significant difference of *P* < 0.5 (*P*-values were adjusted for multiple testing using the Benjamini–Hochberg method). Moreover, these genes proceeded to enrichment analyses in Enrichr (https://maayanlab.cloud/Enrichr/) [55]. For the genes of PRC2, RPKM values were compared between each patient group.

### Mice

WT (C57BL/6N, SLC), *Ezh2*-floxed (obtained from RIKEN BRC: RBRC05555) [56], *Pgr*-Cre [57], and *Ltf*-iCre mice [58] were used in this study. *Ltf*- and *Pgr*-Cre are driven in the endometrial epithelium and the entire uterine layers, respectively [57, 58]. Mice with *Ezh2* deletion in the entire uterine layers (*Ezh2*^*flox/flox*^*Pgr*^*Cre/+*^; *Ezh2* uKO) and in the uterine epithelium (*Ezh2*^*flox/flox*^*Ltf*^*Cre/+*^; *Ezh2* eKO) were generated by crossing *Ezh2*-floxed mice with either *Pgr-*Cre or *Ltf*-iCre mice. Cre-negative littermates (*Ezh2*^*flox/flox*^) were used as controls. All mice used in this study were housed in the University of Tokyo Animal Care Facility following the institutional guidelines in using laboratory animals (approval numbers: P16-066 and P20-076).

### Analysis of pregnancy phenotypes

To analyze pregnancy phenotypes, female mice of *Ezh2* uKO and the littermate control were used at the ages of 7–12 weeks before the obvious increase in epithelial cysts [27, 28]. Females were mated with fertile WT males, and the day on which vaginal plugs were observed was designated as day 1 of pregnancy. Mice were examined for pre-implantation embryo development and implantation on day 4, 5, 6, and 8 as previously described [19, 20, 36]. To examine the pre-implantation development of embryos, mice were sacrificed on day 4 of pregnancy, and the uteri were flushed with saline to recover blastocysts. On day 5 and day 6, ISs were visualized by intravenously injecting a Chicago blue dye solution. In day 8 histology, if no embryo was observed, the embryo was lower than Theiler Stage 8 [59], or if an obvious hematopoietic cell infiltration was observed, they are diagnosed as implantation failures. Parturition events were monitored from day 19 to day 22 by observing mice daily, in the morning and evening.

### H&E staining and immunohistochemistry

H&E staining and immunohistochemistry, using 10% formalin-fixed paraffin-embedded sections (6 μm) or frozen sections (12 μm), were performed as previously described [60]. For immunohistochemistry, Ezh2 (5246, Cell Signaling Technology, Beverly, MA, USA, 1:2000), Ki67 (SP6, Thermo Fisher Scientific, Cheshire, UK 1:400), and CK8 (AB531826, DSHB, Iowa City, IA, USA 1:100) antibodies were used.

### Western blotting

Protein extraction and Western blotting were performed as previously described [61]. Ezh2 (5246, Cell Signaling Technology, 1:100) and actin (sc-1615, Santa Cruz Biotechnology, Dallas, TX, USA, 1:5000) antibodies were used. Bands were visualized under the ECL Prime detection system (GE Healthcare, Chicago, Il, USA). Actin served as a loading control. The full length uncropped original Western blots were shown in the Supplementary information.

### Serum E_2_ and P_4_ level measurement

Blood samples from mice were collected at 1000 h on day 4 of pregnancy. Serum samples were prepared by centrifugation and stored at −80 °C until analysis. Serum E_2_ and P_4_ levels were measured using the Estradiol ELISA kit (ES180S-100, CalBiotech, Spring Valley, CA, USA) and Progesterone EIA kit (582601, Cayman Chemical, Ann Arbor, MI, USA), respectively, according to the manufacturer’s protocols.

### RNA-seq of the mouse uterus

Three uterine tissues/each genotype were collected from the control and *Ezh2* uKO females on the morning of day 4 or 6. For day 4 samples, pseudo-pregnant females were used to preventing the influence on the embryos. The total RNA extraction from mouse uterine tissues was performed using NucleoSpin RNA (MACHEREY-NAGEL, Düren, Deutschland). Collected RNA samples were subjected to RNA-seq using the BGI Japan (Hyogo, Japan) RNA-Seq service following the standard protocol. The resulting raw read files were aligned on the mouse genome sequence (GRCm38/mm10) as performed in “RNA-seq of human endometria.” Genes showing >twofold changes in expression between the control and *Ezh2* uKO with a significant difference of *P* < 0.05 (adjusted *P*-value using the Benjamini–Hochberg method) were classified as DEGs. Upregulated genes in *Ezh2* uKO on days 4 and 6 were subjected to Enrichr [55] for gene enrichment analyses.

### Quantitative RT–PCR analysis

RNA preparation and total RNA extraction from mice uterine tissues were performed using NucleoSpin RNA (MACHEREY-NAGEL). The complementary DNA from the extracted RNA was synthesized using ReverTra Ace qPCR RT Master Mix with gDNA Remover (TOYOBO, Osaka, Japan). qPCR was performed using SYBR Green PCR Master Mix (Thermo Fisher Scientific). The housekeeping gene *Actb* was used as the internal standard to normalize the relative messenger RNA (mRNA) expression. Relative gene expressions were analyzed using the 2^−ΔΔCt^ method [62]. Table S1 shows qPCR primer sequences used to detect each gene.

### Three-dimensional visualization of ISs

As previously reported, three-dimensional (3D) visualization on day 6 ISs was performed [9, 60]. Briefly, an anti-E-cadherin antibody (3195, Cell Signaling Technology, 1:300) was used to stain luminal and glandular epithelia. Alexa Fluor 555-conjugated anti-rabbit antibody (A21428, Thermo Fisher Scientific, 1:300) was used as a secondary antibody. The 3D images were acquired using LSM 800 (Zeiss, Oberkochen, Germany) and AXR (Nikon, Tokyo, Japan). The surface tool in Imaris (v 9.8, Oxford Instruments, Abingdon-on-Thames, UK) was used to construct 3D images.

### Native ChIP

Day 6 uteri from the control and *Ezh2* uKO were used. Uterine tissues surrounding the embryos were longitudinally opened at the mesometrial side and kept at −80 °C until use. Endometrial tissues were disrupted with 35 strokes in a Dounce homogenizer on ice, with a loose-fitting pestle in PBS-containing protease inhibitor cocktail and phosphatase inhibitors 2 and 3 (Sigma, St. Louis, MO, USA). After centrifugation with 1000*g* for 5 min, pellets were received in the nuclei EZ lysis buffer (Sigma) to isolate the nuclei. Native ChIP was then performed as previously described [63], with some modifications. For the fragmentation, chromatins were treated by 20 U/μl MNase at 37 °C for 5 min. Input DNA was analyzed with a 2100 Bioanalyzer system using High-Sensitivity DNA Reagent kit (Agilent, Santa Clara, CA, USA) to confirm DNA fragmentations at approximately 200–300 bps. Chromatin immunoprecipitation was performed using Magna ChIP G-Chromatin Immunoprecipitation Kit (Millipore, Burlington, MA, USA) following the manufacturer’s protocol. Anti-H3K27me3 antibody (39155, Active motif, Carlsbad, CA, USA) or anti-rabbit IgG (2729, Cell Signaling Technology) was used to precipitate the target chromatins. Chromatin-immunoprecipitated DNAs were purified by extracting phenol–chloroforms.

### ChIP-seq

ChIP samples were subjected to ChIP-seq using Novogene Inc. service. The resulting raw read files were aligned on the mouse genome sequence (GRCm38/mm10) using bowtie2 version 2.3.3.1, and peak cells were made using MACS2 [64] with their default settings. Tag density plots and heatmaps in the vicinity were generated using ngs.plot version 2.47.1 [65]. BEDtools intersect [66] (version 2.26.0) was used to compute the numbers of H3K27me3 peaks within ±2 kb around the upregulated genes in *Ezh2* uKO on day 6. Enrichment of H3K27me3 peaks around the target loci was then compared between the control and *Ezh2* uKO with Mann–Whitney *U* test using R (4.0.2). For the analysis focusing on G2M-related cell cycle genes, human cell cycle genes previously reported [67] were converted into mouse gene names using biomaRt in R (4.0.2). To identify the enrichment of known motifs within H3K27me3 enriched loci, we used the HOMER [68] (version 4.9) function findMotifsGenome.pl with default parameters and a fragment size denoted by the argument -gain. To visualize H3K27me3 peaks, the IGVTools count function [69] (Broad Institute, Cambridge, MA) was used to create TDF files from the sorted BAM files. The TDF files were processed in the IGV browser [69] (Broad Institute) to show continuous tag counts over the target loci.

### In vivo bromo-deoxyuridine (BrdU) incorporation assay

In vivo BrdU incorporation assay was performed as previously described [70]. Briefly, female mice were injected with BrdU (100 mg per kg body weight) at 1000 h on day 6 of pregnancy. Two hours later, they were sacrificed and the uteri were frozen immediately. Frozen sections (12 μm) were fixed in methanol for 10 min at room temperature and immersed in 2 N HCl for 20 min at 37 °C to denature DNA for immunohistochemical detection. BrdU-positive area was quantified using Image J (NIH).

### Immunofluorescence

Frozen sections (12 μm) were used for immunofluorescence. BrdU (ab6326, Abcam, 1:250) and phospho-histone H3 (pHH3) (9701, Cell Signaling Technology, 1:300) antibodies were used as primary antibodies. Alexa Fluor 488-conjugated anti-rat immunoglobulin G (A11006, Thermo Fisher Scientific, 1:300) and Alexa Fluor 555-conjugated anti-rabbit immunoglobulin G (A21428, Thermo Fisher Scientific, 1:300) were used for signal detection with nuclear staining using 4,6-diamidino-2-phenylindole (Dojindo, Kumamoto, Japan 1:500). Images were obtained using LSM 800 (Zeiss) and AXR (Nikon). The decidual area without pHH3 staining was quantified by Image J (NIH).

### Senescence-associated β-galactosidase (SAβgal) staining

To evaluate the terminal differentiation of decidua, SAβgal activity staining was performed as previously described [35, 36]. To compare the intensity of SAβgal staining between the control and *Ezh2* uKO, frozen sections from both genotypes were processed on the same slide. Sections were counterstained with eosin.

### Statistical analysis

Statistical analyses were performed using two-tailed Student’s *t*-test in GraphPad Prism 9 (GraphPad Software, San Diego, CA); otherwise, they are described in detail in each experimental section.

## Supplementary information

Table S1

Supplementary Figure Legends and Supplementary Data Legends

Figure S1

Figure S2

Figure S3

Data S1

Data S2

Data S3

Original Western blots for Figure 2C

Reproducibility Checklist

## Data Availability

The data of RNA-seq and ChIP-seq were deposited in Gene Expression Omnibus (The accession number: GSE207362). Among the uploaded human RNA-seq data, we only used the one from patients aged under 40 years without uterine diseases: #2, 3, 5–7, 9–13, 16–18, 20, 21, 23, 24, 26, 31–33, 35, 36, and 38–40 for fertile control; #1, 4–7, 9, 14, 17, 19, 21, 23, and 25 for RIF.
