# Supplementary material for: The EZH2–PRC2–H3K27me3 axis governs the endometrial cell cycle and differentiation for blastocyst invasion
Source: Cell Death Dis. 2023 May 18;14(5):320. doi: 10.1038/s41419-023-05832-x (PMC10192223; doi:10.1038/s41419-023-05832-x)
Supplement: Supplementary file 2 — Supplementary Figure Legends and Supplementary Data Legends [file 41419_2023_5832_MOESM2_ESM.docx]

**Supplementary Figure Legends.**

**Figure S1. Immunostaining of EZH2 in the human endometrium, provided by the Human Protein Atlas.** Representative images of EZH2 staining in the human endometrium. Tissues from patients of reproductive ages (35 and 42 years old) and elderly ages (61 and 72 years old) were shown.

**Figure S2. *Ezh2*-deletion affects cell proliferation-related and E_2_-responsive genes in the mouse uterus on day 4.** (A) RNA-seq was performed on day 4 uteri (n = 3 mice for each group). Significantly up-regulated genes (95 genes) in *Ezh2* uKO mice are highlighted by magenta and down-regulated genes (61 genes) in *Ezh2* uKO mice are highlighted by turquoise. (B) Gene enrichment analyses by Enrichr revealed the up-regulated genes had high enrichment in cell proliferation and estrogen signaling pathway. (C) RT-qPCR results of E_2_-responsive genes (*Muc1* and *Ltf*) and P_4_-responsive ones (*Ihh* and *Areg*) in *Ezh2*-deleted uteri on day 4. Data are mean ± SEM, *P* values by Student's t-test. At least n = 3 for each group.

**Figure S3. Increased levels of lncRNAs in *Ezh2*-deleted endometria on day 6.** (A) Among the up-regulated genes in day 6 *Ezh2* uKO uteri (Figure 6A), lncRNA genes were highlighted. (n = 3 mice for each group). (B) Reads per kilobase of transcript per million mapped read (RPKM) values of the up-regulated lncRNAs were shown.

**Supplementary Data Legends.**

**Data S1. DEGs between the human peri-implantation endometrium of the RIF patients and the fertile controls.** Each data set was processed into DESeq2 to identify DEGs which have at least 2-fold expression changes with adjusted *P*-values < 0.5.

**Data S2. DEGs between the mouse endometrium of the *Ezh2* uKO and the control females on day 4 of pregnancy.** Each data set was processed into DESeq2 to identify DEGs which have at least 2-fold expression changes with adjusted *P*-values < 0.05.

**Data S3. DEGs between the mouse endometrium of the *Ezh2* uKO and the control females on day 6 of pregnancy.** Each data set was processed into DESeq2 to identify DEGs which have at least 2-fold expression changes with adjusted *P*-values < 0.05.
