# Supplementary figures and images for: The EZH2–PRC2–H3K27me3 axis governs the endometrial cell cycle and differentiation for blastocyst invasion
Source: Cell Death Dis. 2023 May 18;14(5):320. doi: 10.1038/s41419-023-05832-x (PMC10192223; doi:10.1038/s41419-023-05832-x)

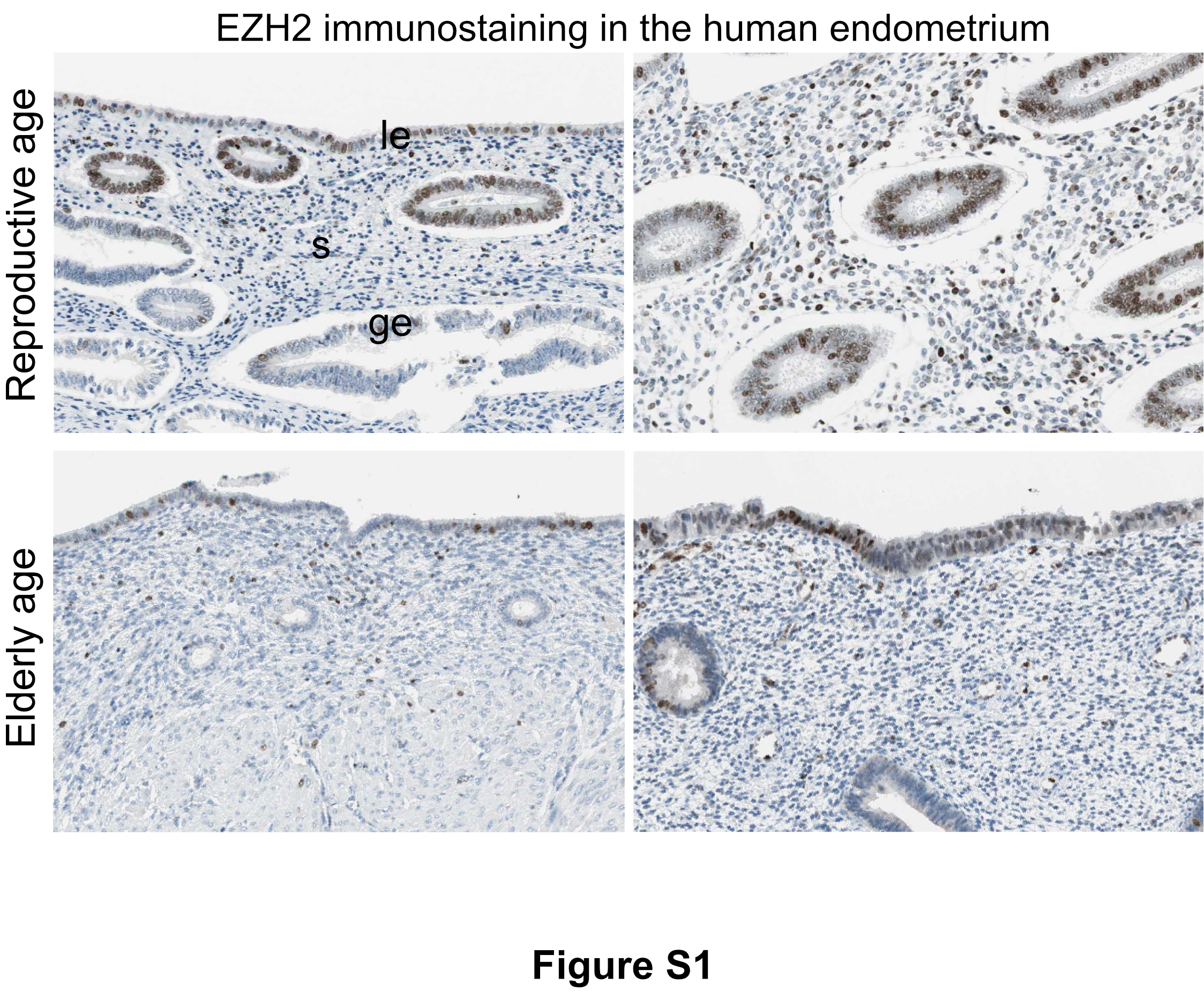

Supplement: Supplementary file 3 — Figure S1 [file 41419_2023_5832_MOESM3_ESM.tif]

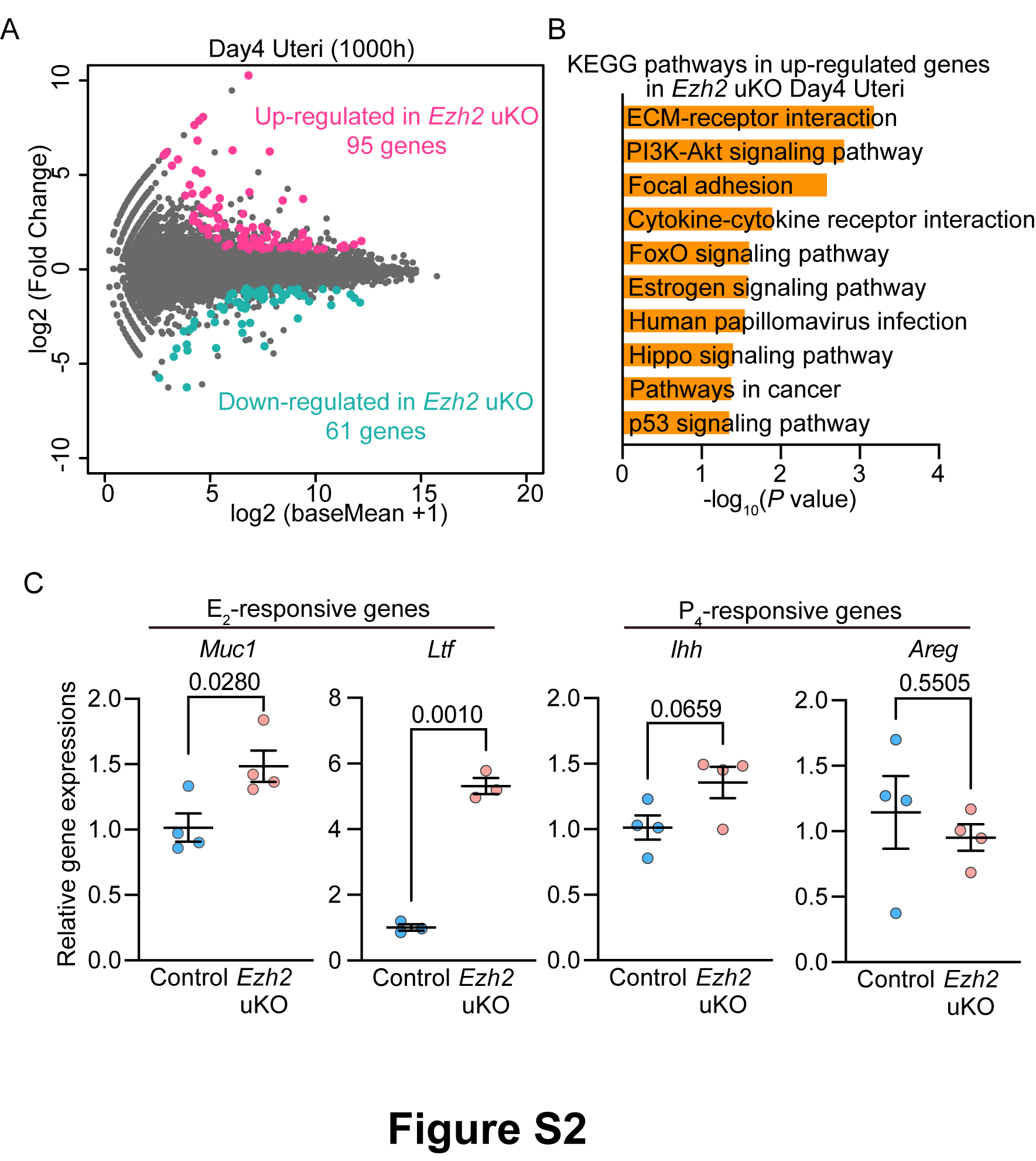

Supplement: Supplementary file 4 — Figure S2 [file 41419_2023_5832_MOESM4_ESM.tif]

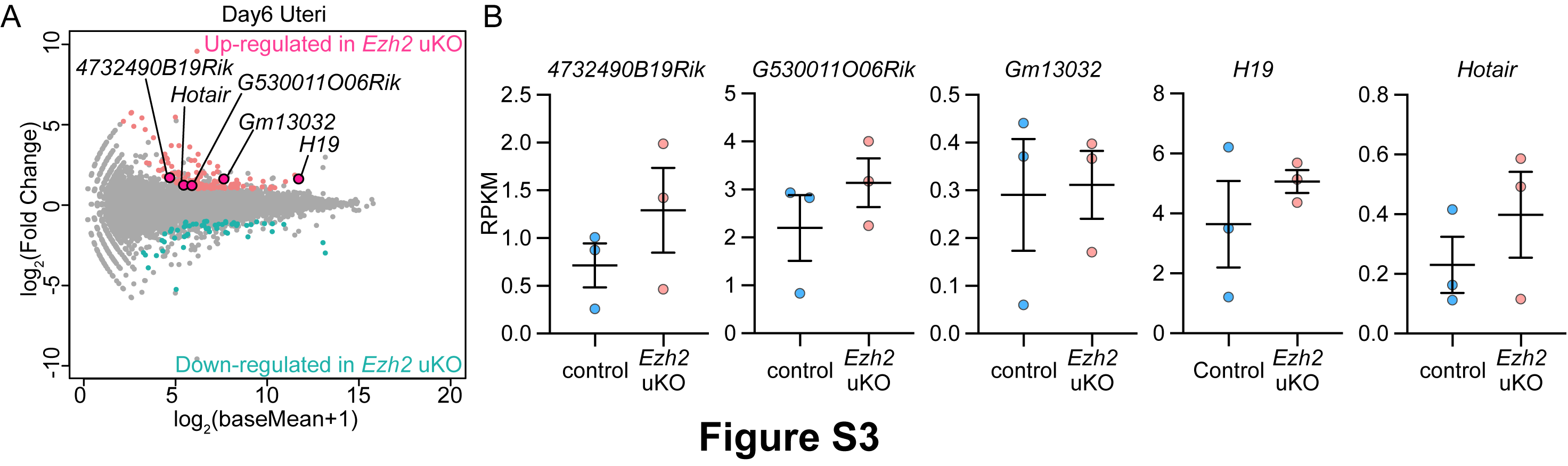

Supplement: Supplementary file 5 — Figure S3 [file 41419_2023_5832_MOESM5_ESM.tif]

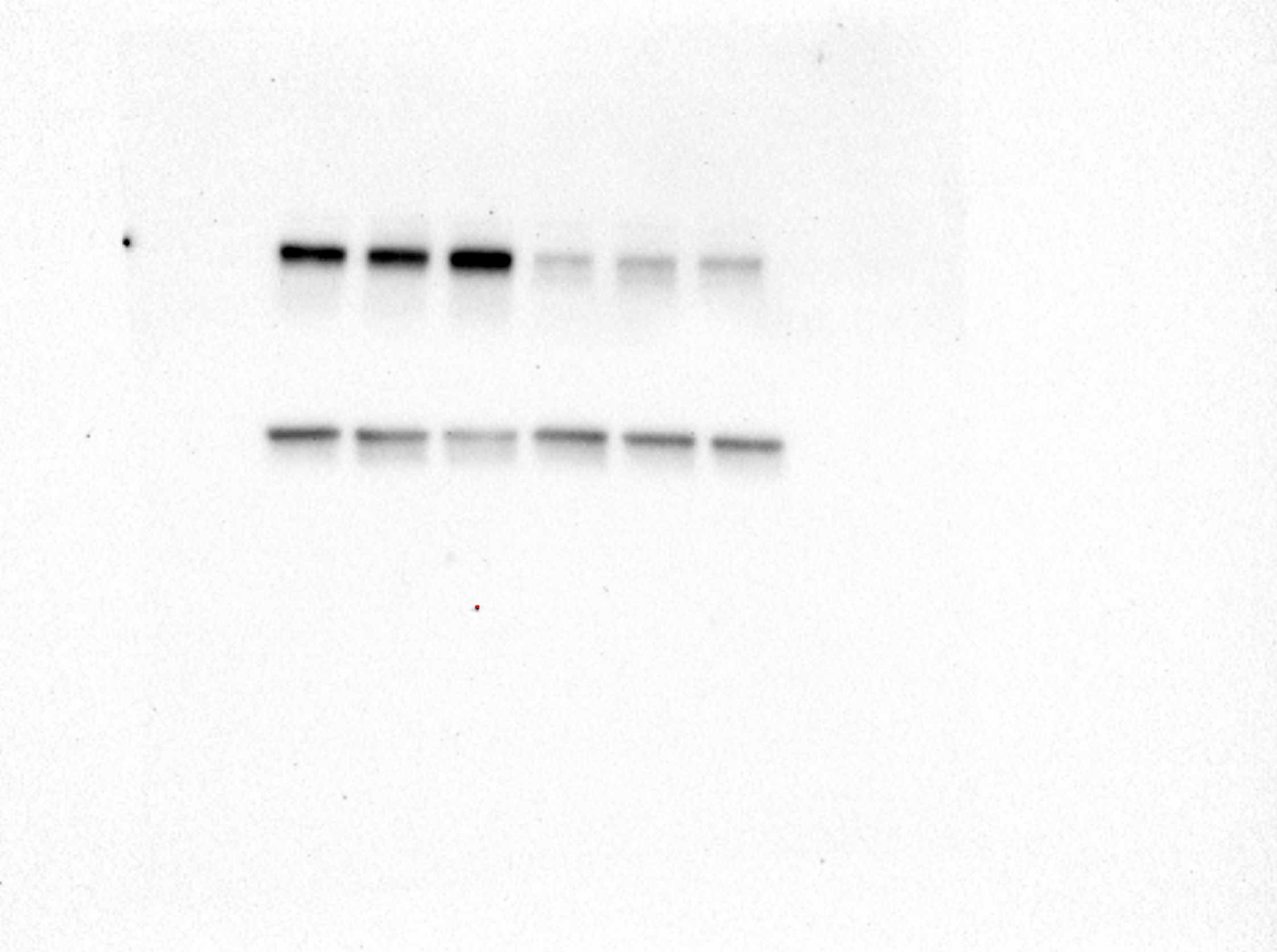

Supplement: Supplementary file 9 — Original Western blots for Figure 2C [file 41419_2023_5832_MOESM9_ESM.tif]
